# “*I think there has to be a mutual respect for there to be value*”: Evaluating patient engagement in a national clinical trial on de-implementation of low value care

**DOI:** 10.1186/s40900-023-00483-w

**Published:** 2023-08-26

**Authors:** Holly Etchegary, Stefanie Linklater, D.’Arcy Duquette, Gloria Wilkinson, Vanessa Francis, Erin Gionet, Andrea M. Patey, Jeremy M. Grimshaw

**Affiliations:** 1 Clinical Epidemiology Program, Faculty of Medicine, Patient Engagement Lead, NL SUPPORT, CIHR-SPOR, Craig L. Dobbin Centre for Genetics, Memorial University, 300 Price Phillip Parkway, Rm 4M210, St. John’s, NL A1B 3V6 Canada; 2 Department of Medicine, Centre for Implementation Research, Ottawa Hospital Research Institute, University of Ottawa, Ottawa, Canada; 3 Patient Partnership Council of the De-Implementing Wisely Research Group, Calgary, Canada; 4 Owerko Centre, Alberta Children’s Hospital Research Institute, University of Calgary, Calgary, Canada

**Keywords:** Patient engagement, Evaluation, De-implementation, Low value care, Trial, Choosing Wisely

## Abstract

**Background:**

The evaluation of patient engagement in research is understudied and under-reported, making it difficult to know what engagement strategies work best and when. We provide the results of an evaluation of patient engagement in a large Canadian research program focused on the de-implementation of low-value care. We aimed to evaluate the experience and impact of patient engagement in the study.

**Methods:**

An online cross-sectional survey was administered using Microsoft Forms to (1) researchers and study staff and (2) patient partners. The survey was developed following iterative reviews by the project’s patient partnership council and evaluation committee. Survey content areas included opinions on patient engagement to date, including challenges to engagement and suggestions for improvement. Patient partners also evaluated the partnership council. Descriptive statistics including counts and percentages described Likert scale survey items, while open comments were analyzed using descriptive content analysis.

**Results:**

The survey response rate was 46% (17/37). There were positive attitudes about the value of patient engagement in this project. There was also a high degree of willingness to be involved with patient engagement in future projects, whether as a patient partner or as a researcher including patients on the research team. Most patient partners felt their contributions to the project were valued by researchers and study research staff. Open comments revealed that a co-design approach and full inclusion on the research team were integral to demonstrating the value of patient partner input. Areas for improvement included more frequent and ongoing communication among all team members, as well as earlier training about patient engagement, particularly addressing role expectations and role clarity.

**Conclusions:**

Our data revealed that despite some challenges, team members recognized the value of patient engagement in research and agreed project decisions had been impacted by patient partner input. Ongoing communication was highlighted as an area for improvement, as well as earlier training and ongoing support for all team members, but particularly researchers and study staff. In response to evaluation data, the team has reinstated a quarterly newsletter and plans to use specific patient engagement planning templates across study sites for all project activities. These tools should help make expectations clear for all team members and contribute to a positive patient engagement experience. Findings can inform patient engagement planning and evaluation for other health research projects.

**Supplementary Information:**

The online version contains supplementary material available at 10.1186/s40900-023-00483-w.

## Abstract (plain language summary)

Evaluating patient engagement in research is often not done or not reported, making it hard to know what engagement strategies work best and when. Here, we provide the results of an evaluation of patient engagement in a Canadian Institutes for Health Research (CIHR) Strategy for Patient Oriented Research (SPOR) Innovative Clinical Trial Multi-Year Grant. The project focuses on strategies to reduce two low-value care practices (pre-operative testing in low-risk day surgery and imaging for low back pain). An online survey was sent to project researchers, study staff and patient partners to get their opinions on the patient engagement in the project. Generally, there were positive attitudes about the value of patient engagement in the project. Both patient partners and research study staff were very willing to be involved with patient engagement in future projects. Most patient partners felt their contributions to the project were valued by researchers and study research staff. An important part of showing the value of patient partners was working together to design the project and making sure that the patient partners were considered full members of the research team. Areas for improvement included better communication among all team members and earlier training about patient engagement with a focus on patient roles and expectations. The results from this survey will be used to improve the patient engagement in this project but will also help patient engagement planning and evaluation for other health research projects.

**Supplementary Information:**

The online version contains supplementary material available at 10.1186/s40900-023-00483-w.

## Background

Partnering with patients about their lived experiences of health and illness and their experiences in the healthcare system can help inform the provision of care, health policies and health research with a goal to improve outcomes. A growing literature provides methods, best practices, and guidance for research teams on how to engage patients in research [1–7]. Evidence suggests that partnering with patients in the design and conduct of health research can improve both research quality and outcomes [8–10].

However, the evaluation of patient engagement in research is understudied and under-reported, making it difficult to know what engagement strategies work best and when [5, 10, 11].

Reports on patient engagement experiences largely come from individual study teams or projects [12], and highlight promising practices for (and challenges to) engagement. Challenges often revolve around infrastructure barriers (e.g., time, resources and communication practices needed to support engagement). Challenges are also noted in relationship building (e.g., recruiting a diversity of patient partners) and maintaining relationships with patient partners (e.g., ensuring true inclusion of patient partners and real influence on project decisions) [13]. The lack of robust evaluations of patient engagement in research projects can fuel the criticism of patient engagement cynics, but also hinder the work of those who support it and wish to regularly improve their engagement practices [14]. While multiple frameworks for the evaluation of patient and public engagement now exist, there is little consensus on what indicators and outcomes should be included in such evaluations, and a co-development approach to evaluation with patient collaborators in local contexts has been suggested [11, 14, 15].

In this report, we present the results of a co-developed evaluation of patient engagement in a Canadian Institutes for Health Research (CIHR) Strategy for Patient Oriented Research (SPOR) Innovative Clinical Trial Multi-Year Grant (GRANT # MYG-158642).

## Methods

### Evaluation context

This evaluation uses a cross-sectional survey to explore patient engagement in a multi-provincial research program. The De-implementing Wisely Research Program, is guided by the Choosing Wisely De-Implementation Framework [16], and partners with Choosing Wisely Canada, Choosing Wisely provincial campaigns, patients, researchers and health system partners in three Canadian provinces (Alberta, Newfoundland & Labrador, and Ontario). The program aims to design theory informed de-implementation interventions targeting both healthcare professionals and patients to reduce two low-value care practices (pre-operative testing in low-risk ambulatory surgery and imaging in uncomplicated low back pain) and evaluate them using cluster randomized trials (CRTs) (giving a total of six individual CRTs). While the COVID 19 pandemic necessitated revised program activities and a reduction in the number of CRTs, the program retained a strong focus on patient engagement.

### Patient engagement in the de-implementing Wisely research program

Three provincial lead patient partners were recruited as co-investigators at the time of grant writing and contributed to the program design and final application. After the research project received funding, two additional patient partners were recruited from each province giving a total of nine patient partners across the three provinces. Upon their suggestion, a pan-Canadian patient partnership council (PPC) was established as the key mechanism of patient engagement for the study. Recruiting for and establishing the PPC took a little over a year. Since its creation in 2019, three members left due to moves or other commitments, with new members recruited as needed. There are seven current council members.

The project provided a number of supports to patient partners including a Patient Engagement Coordinator (a part time project-employed team member responsible for coordinating PPC meetings and activities and acting as a key liaison among patient partners and other team members (EG, currently SL), a Scientific Patient Engagement lead (a co-investigator with extensive experience of supporting and researching patient engagement activities (HE) and the overall Program Coordinator (AMP)); these team members served as non-voting members on the PPC.

PPC members collaboratively developed a Terms of Reference for the group through an iterative process over multiple PPC meetings and reviews of drafts by all council members. It is reviewed annually. The Terms of Reference outlines an appreciation policy for the remuneration of patient partners that is standard across study sites. Further, the council’s role in the project overall and specific patient engagement activities (e.g., providing early presentations to the large research team, contributing to the project newsletter, creating knowledge translation tools such as infographics about patient engagement) are also outlined. Notably, the PPC prioritized the evaluation of the patient engagement strategies and activities over the course of the project and evaluation is included in the Terms of Reference. The COVID 19 pandemic delayed the evaluation of patient engagement as many study activities were paused or altered. This report follows the GRIPP2 short reporting checklist [17] (Additional file 1) and describes the first evaluation of patient engagement in this large research program.

### Evaluation planning process

While the pandemic delayed the collection of evaluation data, evaluation planning was the focus of two dedicated PPC meetings in late 2020 and early 2021. Detailed discussions considered whose views should be elicited (both patient partners and researchers/study research staff) and how (an online survey). Three members of the PPC (DD, GW, VF) along with the patient engagement lead and coordinator (HE, SL) formed an evaluation planning committee. This team met as needed to draft and finalize evaluation instruments and the data collection process.

### Evaluation invitation criteria

The evaluation planning committee, with input from the Project Principal Investigator (JMG) and the research lead (AMP) reviewed a list of the program co-investigators and collaborators to determine those who had the opportunity to date to engage with the national or provincial teams on study activities. Investigators whose expertise had not yet been required for the project (e.g., intervention development, knowledge translation) were not invited to evaluate patient engagement as they had no interaction with patient partners to date.

### Evaluation instrument

The evaluation planning committee reviewed existing evaluation instruments outlined in the Centre of Excellence on Partnership with Patients evaluation toolkit [18], as well as instruments provided by patient partners used in their other patient engagement activities to select and create items for the current evaluation. Two online surveys—one for patient partners and one for researchers/study research staff -with a mix of Likert-scale and open items were developed. The patient partner survey contained sections on patients’ motivation for becoming a patient partner, opinions on engagement to date in the project, opinions on the PPC specifically and any suggestions for patient engagement in the remainder of the project (N = 22 items). The researcher/study research staff survey was similar but did not include the sections on patient partner motivations or opinions on the PPC (N = 14 items). Demographic information was not collected excepting province of residence due to anonymity concerns. The final survey instruments were the result of iterative reviews by the evaluation planning committee and the larger PPC, who approved final survey versions. No formal pretesting of the evaluation surveys was completed. See Additional file 2 for full instruments.

### Survey administration

Respondents were invited by e-mail by the Patient Engagement Coordinator (SL) to complete the evaluation survey. The survey was hosted online using Microsoft Forms and was open for approximately one month. An initial email was sent in June 2022 with a follow up email one week later. A second reminder email was sent in July 2022 a week before the survey closed. Once the survey closed, responses were exported into Excel for analysis.

### Survey analysis

Descriptive statistics including counts and percentages were used to describe closed, Likert scale survey items. Open-ended survey items were analyzed using constant comparison and qualitative description, given the goal was to provide a comprehensive description and summary of respondent comments [19, 20]. Answers to open items were placed in a Microsoft Excel table where respondent comments were first read and re‐read independently by two team members (SL, HE) to begin identifying emerging codes and ideas [19]. A formal codebook was not developed; however, discussion between the analysts following the coding of the open comments in the first five surveys revealed very similar codes emerging. Once independent coding of all survey open items was complete, investigators met again to discuss coding decisions. Differences in coding tended to be minor wording issues and were resolved through discussion. Patient partners on the evaluation planning committee subsequently reviewed codes and representative quotes; no further changes or questions arose following their review.

### Ethics

The Ottawa Health Science Network Research Ethics Board advised ethics review was not required for this work as it was an evaluation, rather than a research project.

## Results

### Response rate

The overall response rate was 46% (17/37). Seven of eight patient partners completed surveys (87.5%), while 10/29 (34%) researchers/study research staff returned surveys. In what follows, we present patient partner evaluation data, followed by data from researchers/study staff; in the final section, we present data from the open-ended survey items asked of both groups of respondents. Quotes from respondents are identified only by whether they are a patient partner or a researcher/research study staff (with de-identified study ID # in brackets) and by Province 1, 2, or 3 to help protect respondent anonymity.

Patient partner evaluation data.

#### Motivations for becoming a patient partner

Patient partners were motivated to join the research program because they felt their lived experiences could contribute to healthcare improvements and patient outcomes, a key goal of this project.

To have a voice in creating safety and efficiencies in our healthcare through lived experience discussions. - Patient Partner (ID# 1), Province 1

Lived experience with lower back pain and an awareness of low value care coupled with dangers of excessive imaging made study relevant to me. Especially attracted to potential practical applications. – Patient Partner (ID# 7) Province 2

#### Reflections on the patient partnership council

**Fig. 1:**
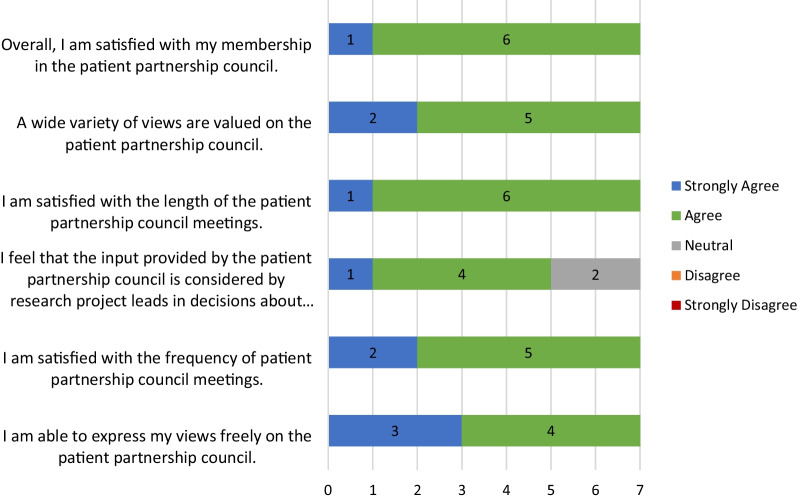
Attitudes towards the patient partnership council

Patient partners were satisfied with their membership in the council, agreeing with the frequency and length of meetings and their ability to express their opinions (Fig. 1). All agreed or strongly agreed that a wide variety of views were valued on the council.

However, at least two patient partners were unsure about how council feedback was considered by project leads. As one noted:

Communication with provincial leads has not been strong making it difficult to really evaluate how the input has been received and the actual impact patient partner participation has impacted the research. - Patient Partner (ID# 7) Province 2

#### Opinions on engagement in the research project

**Fig. 2:**
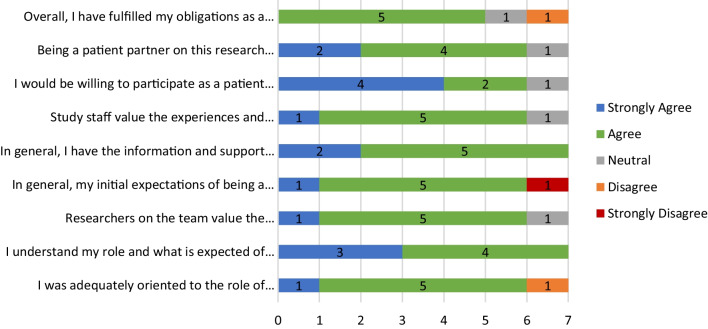
Patient partner attitudes towards patient engagement

Patient partners saw their participation as a good use of their time and would be willing to be a patient partner on future research projects (Fig. 2). Most (6/7) also felt that researchers and study staff valued their suggestions. While most patient partners understood their role and felt they were sufficiently oriented to the role of patient partner on the project, a minority did not feel adequately oriented and indicated their expectations of being a patient partner were not met (Fig. 2). All patient partners agreed or strongly agreed they had the information and support they needed to engage with the project.

#### Patient partner reflections-The good, the bad, and ongoing motivation

Patient partners indicated that engaging with their peers in an inclusive environment was an important part of their engagement.

The people I have met. – Patient Partner (ID# 1) Province 1.

The feeling of inclusion has been really important to me.—Patient Partner (ID# 2) Province 2.

Met other patients with common values but diverse backgrounds and approaches. Staff that have been dedicated to making patient experience a positive one. - Patient Partner (ID# 7) Province 2

Patient partners also enjoyed learning about the research process.

*Sharing opinions and learning about how research is done.*—Patient Partner (ID# 3) Province 3.

It has been good to be able to have discussions with patient partners and researchers from other provinces, and learn about different perspectives. Also have enjoyed some of the model development and involvement within our provincial group. - Patient Partner (ID# 5) Province 2

Patient partners noted two consistent challenges to their engagement: COVID 19 and communication. As in many research projects, the COVID 19 pandemic caused significant changes and delays.

Covid has certainly caused slowdowns in the research project, and has challenged progress. - Patient Partner (ID# 5) Province 2

I think we try but we still have communication gaps and sometimes the PP don't know what's going on. – Patient Partner (ID# 1) Province 1

Delays due to COVID 19; inconsistent communication; staff/leadership turn over. - Patient Partner (ID# 7) Province 2

Patient partners provided suggestions for the research team on what would help keep them motivated to engage with the project. Ongoing and repeated communication and interaction with other research team members was noted as critical:

There has to be a connection with the researchers on an ongoing basis, and continued consultation. - Patient Partner (ID# 5) Province 2

Continuing to be updated on the project and being able to share our thoughts and ideas keeps my motivation in this research. - Patient Partner (ID# 2) Province 2

More discussions and more opportunities. Lots going on in the system and patients can play a key role in positive changes. - Patient Partner (ID# 1) Province 1

Seeing the results of research translated into practice was also noted as a key motivator for ongoing patient engagement.

*Work translates into positive changes.*—Patient Partner (ID# 3) Province 3.

*Plan that sees actual clinical trials implemented.*—Patient Partner (ID# 7) Province 2.

Researcher/study staff evaluation data.

**Fig. 3:**
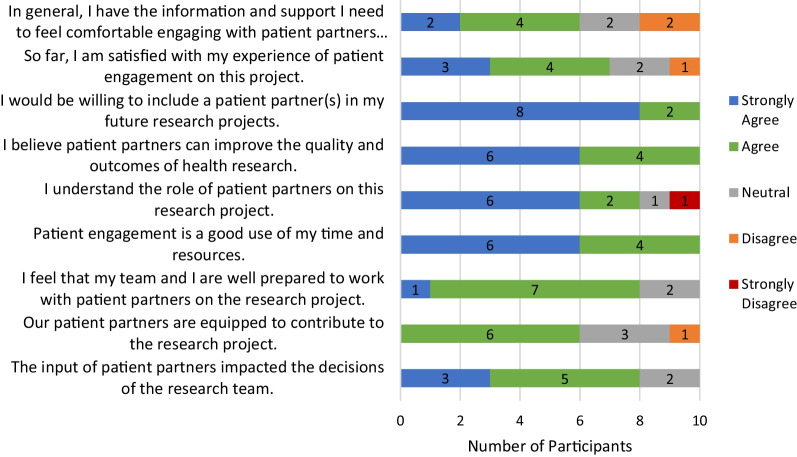
Researcher/study research staff attitudes towards patient engagement

Figure 3 displays researcher/study research staff attitudes towards patient engagement in the project. In general, researchers and study staff agreed (7/10) they were satisfied with their experience of patient engagement on the project and all agreed (most strongly) they would be willing to engage patient partners in future projects. All agreed or strongly agreed that patient partners can improve health research and patient engagement was a good use of resources.

However, a sizeable minority (4/10) indicated they did not have adequate information or support to feel comfortable partnering with patients on this project. While most agreed patient partner input impacted research decisions, some were unsure or disagreed that patient partners were equipped to contribute to the research (Fig. 3). Similarly, while most researchers and study staff understood the role of patient partners (8/10), a minority did not or were unsure. Open comments highlighted both the importance of understanding expectations and opportunities for training:

New staff could have a training session before collaborating with patient partners - Researcher/ Study Research Staff (ID# 3) Province 3

It would be helpful to have support/training on how to deal with PP expectations or how to better incorporate them into the research process. - Researcher/ Study Research Staff (ID# 7) Province 1

I think the challenge for me is balancing the Patient expectation with the expectation of the research team and the project overall. Sometimes they do not overlap at all and I struggle to keep everyone happy - Researcher/ Study Research Staff (ID# 4) Province 2

Open-ended evaluation items asked of both patient partners and researchers/study staff.

#### Tangibly demonstrating the value of patient partners

All respondents were asked, “How can researchers/study staff show that they value patient partner contributions?” in an open-ended item. Patient partners indicated that the value of their involvement could be demonstrated by other study team members through ongoing communication, active listening and a commitment to the values of inclusion and respect by fully incorporating patients as part of the team.

The art of listening is so important when involving patient partners. Including us in as much or as little as we want in the project has been fabulous! I enjoy being updated and included in every aspect of the process. All these things make me feel valued. - Patient Partner (ID# 2) Province 2

Listening, identifying how patient partner contributions contribute to the quality of the research. Including patient partners as part of the team. – Patient Partner (ID# 5) Province 2

Respect. Full inclusion. –Patient Partner (ID# 4) Province 1

Researchers and study research staff agreed that showing value included communication and full inclusion of patients as team members, but added the importance of listening to one another and respecting different perspectives, while taking into consideration the overall goals of the study.

I think there has to be a mutual respect for there to be value. I think that people on the team may have their own agenda, but ultimately the outputs of the project are dictated by the grant. I think both the patient partners and the researchers have to listen to one another and respecting the different perspectives but remember the goals of the project. – Researcher/Study Research Staff (ID# 4) Province 3

I think keeping them informed and involved where appropriate/makes sense is the best way to show that they are valued. I think treating them as the exception or less than/different to any other researcher/research staff is a disservice to the value they bring to the research itself. Sometimes I still hear them qualify the "research team" with including patient partners, but the point is that they are part of the research team, period. - Researcher/Study Research Staff (ID# 1) Province 2

Showing patients where their contributions have made a change/reinforced our approach. So not just the end product (here's what we accomplished) but the nuts and bolts of how/what they contributed lead to those accomplishments. – Researcher/Study Research Staff (ID# 9) Province 2

Researchers and study research staff also highlighted the importance of recognizing contributions made by patients through publications (authorship) and financial reimbursement.

When we acknowledge their contributions in any publication, poster, etc as co-authors. Not only in the acknowledgements section. – Researcher/Study Research Staff (ID# 3) Province 3

They get asked their thoughts and ideas - I prefer to ask them first versus last. Their contributions are acknowledged and depending on the grant allowance, they are paid for major work that they do. – Researcher/Study Research Staff (ID# 5) Province 1

#### The impact of patient partners

Respondents were asked to describe any impacts they had seen in the project as a result of patient partner input. Patient partners indicated positive impacts as they felt that researchers had learned from them and better understood the value that they bring to the research team. They also felt that being a part of the study allowed patient partners to realize their own potential.

I think the researchers have a much better understanding on patient partners and the value they bring to the table. – Patient Partner (ID# 1) Province 1

More people understand the potential/value of patient partner participation in research. Patient partners have grown to better appreciate their potential. - Patient Partner (ID# 7) Province 2

Researchers/study research staff described numerous impacts throughout the research process. The following quote is illustrative of the range of impact:

The patient partners have, in my mind had a tremendous impact - we've added projects based on their comments, they've helped advise on recruitment strategies, reviewed results from projects, helped us interpret findings from the patient perspective, and they have developed materials for dissemination. They've also been pivotal in helping develop the engagement plan template (see ref [26]) which we've used across the projects. – Researcher/Study Research Staff (ID# 4) Province 3

However, the impact of patient partner input may not be consistent across study sites. For example:

The infographics were great, I really liked those together with the patient engagement plans. So far, provincially, I haven't perceived positive impacts. On the contrary, it feels like more effort is needed to accommodate them rather than facilitating interactions/outputs. While this is not true for all, I feel experiences in the other provinces have been more positive and have led to more outputs. – Researcher/Study Research Staff (ID# 8) Province 1

#### Defining successful engagement

Survey respondents indicated that the definition of successful patient engagement centered around full and equal inclusion of patients as study team members with their voices being heard.

Successful patient engagement is when patient partners feel at ease with sharing their views with the team. The sense of inclusion is essential. Knowing what our responsibilities are throughout the project has been a great tool! - Patient Partner (ID# 2) Province 2

That the patient partners felt their opinions, ideas were heard, they were treated as equal members of the team and they actively participated on the activities within the grant. – Researcher/Study Research Staff (ID# 5) Province 1

I think successful patient engagement means you have created a research team that includes patient partners as valid equal members and as a result the product/s of your research have successfully and appropriately included the patient perspective. - Researcher/Study Research Staff (ID# 1)Province 2

For patient partners in particular, it was noted that inclusion of the patient perspective must cover the full spectrum of the research cycle/project. As one noted, successful patient engagement is defined by “*Patient involvement from the start of the planned study until the end of the study.”* – Patient Partner (ID# 6) Province 1.

Continued, impactful engagement was also part of success.

Progressively more patient involvement with more members of the research team at more impactful levels. – Patient Partner (ID# 7) Province 2

There was also a sense of wanting to carry lessons learned beyond the current project.

I see it as a two pronged approach; 1) deliverables at the end of the project that patients could look at and say, proudly, "I contributed to that project, I helped out on that." and Researchers saying "Patients really improved the outputs developed in this project" and 2) conceptually, we were able to contribute to improving how research teams and patient partners engage with one another after this project ends. – Researcher/Study Research Staff (ID# 4) Province 3

#### Suggestions for improving the patient engagement experience

Suggestions from patient partners for improving the patient engagement experience included a range of considerations:

*Orientation to the role—expectations, *etc*.*—Patient Partner (ID# 3) Province 3.

Keep us more involved, ask us questions and avoid assumptions. - Patient Partner (ID# 6) Province 1

More consistent communication especially at the national level - especially important as result of COVID 19 interruptions/delays. Revisit appreciation guidelines with greater flexibility especially at provincial level. - Patient Partner (ID# 7) Province 2

Suggestions from researchers and study research staff for improving the patient engagement experience included communication, funding for patient engagement and more opportunities for patients to present results and provide input.

I think better communication always helps but I think the communication right now is working fairly well. Just always room for improvement. - Researcher/Study Research Staff (ID# 1) Province 2

Now that we have the ability to train patient partners as researchers, it would be great if we could set aside some funds to train nationally. - Researcher/Study Research Staff (ID# 5) Province 1

I think if there was a venue and expectation for all new projects to be pitched and a patient engagement planning meeting facilitated by the PPC, maybe that would be helpful? - Researcher/Study Research Staff (ID# 2) Province 2

Would be great if a patient partner representative could give a presentation or submit an abstract at a research conference, reporting their involvement in the study or patient partner perspectives - Bring other ideas that we did not consider. - Researcher/Study Research Staff (ID# 3) Province 3

## Discussion

This paper describes an evaluation of patient engagement in a large national research program in Canada from both patient partner and researcher/study staff perspectives. Overall, there were positive attitudes about the value of patient engagement in research, in line with literature [1, 4, 8, 11]and other project evaluations of patient engagement [11, 21]. There was also a high degree of willingness to be involved with patient engagement in future projects, whether as a patient partner or as a researcher including patients on the research team, boding well for the sustainability of patient engagement and co-designed health research.

Most patient partners felt their contributions to the project were valued by researchers and study research staff, a motivator of ongoing engagement and satisfaction [11, 13, 21]. Data revealed that a co-design approach and full inclusion on the research team were integral parts of demonstrating the value of patient engagement, as well as components of ‘successful’ patient engagement. These data are in line with the values of inclusion and mutual respect that underline Canada’s Strategy for Patient Oriented Research [22], as well as best practices for patient engagement [2, 7, 11, 13].

Researchers and study staff also suggested that value could be demonstrated by recognizing contributions made by patients through publications (authorship) and financial recognition. It is notable that these were not cited by patient partners, suggesting these may not be as important as feeling respected and included as an equal team member. However, both compensation for patient engagement and authorship are important topics in the literature and guidance exists for both [23–25]. Findings suggest it would be valuable to have early discussions within research teams about both issues to ensure expectations are met (of both researchers and patients) and patient partners feel included and heard in these discussions. In this project, budgeting for patient engagement was recommended by one of the lead patient partners at the time of grant writing; the council also co-created an appreciation policy. These early discussions within the project team and a tangible remuneration policy likely contributed to the lack of concerns from patient partners around compensation. We, and others [24, 25], strongly support budgeting for patient engagement, including appreciation, but acknowledge that not all patient partners will have the same preference for compensation (or authorship) and recommend specific discussions with each project patient partner around these issues.

All patient partners agreed they had the information and support they needed to engage in the project, in line with best practice recommendations for patient engagement [2, 6, 8]. However, a sizeable minority of researchers/study research staff (4/10) indicated they did not have adequate information or support to feel comfortable partnering with patients on this project, and at least one patient partner suggested they did not feel adequately oriented to their role in the project. Greater role clarity has been suggested by other patient and family advisory members in research as an area for improvement [12, 13, 21]. These findings and ours suggest early training and resources would be useful to prepare research teams for engagement. In this project, the development of specific and tangible patient engagement plans [26] were required in order to allow all relevant team members to clearly understand their roles and responsibilities for each project activity. This was especially important as project plans shifted due to COVID 19 and new activities were added. Templates such as ours [26] can be used widely by other health researchers and patient partners, customized to fit any specific project, and we recommend creating these at the beginning of a research project. In our project, these were not created early; their need became obvious only after over many months of the project as team members were challenged to really understand and implement patient engagement. While the PPC had co-designed a Do’s and Don’ts of Patient Engagement infographic (see Additional file 3) early in the project and distributed this to the team, it was not specific enough to fully describe team members’ roles. In contrast, the patient engagement planning templates were province-specific and fully explained all project activities and team member roles.

While most patient partners felt their expectations were met, at least one didn’t and some open comments also revealed the importance of understanding expectations of all team members, particularly related to the funded project. Challenges arose when expectations didn’t match or expected activities were beyond the scope of the funded project. These findings highlight how important early communication is about the scope of a project and a fulsome discussion about what is (or is not) possible within the confines of a specific research project. Nearly all respondents offered more or ongoing communication as an important suggestion for improvement and frequent communication opportunities offer one way of clarifying expectations as a project progresses.

Relatedly, several patient partners noted the importance of moving findings into practice and this being a motivator for their continued engagement. This finding highlights the importance of early and ongoing discussions among the research team as to how project interventions will be moved into practice and whether they will be sustained beyond the life span of the research program. The translation of research findings into practice is challenging [27], as is the sustainability of research project interventions such as ours into clinical care. This may surprise some team members, especially if it is a first time engaging as a partner in a research project. The complexity of scaling up and sustaining project interventions beyond a research project can bring about frustration and as our findings suggest, impact patient partner motivation for continuing in a research project. Again, early and honest discussion about project goals, outputs and knowledge translation activities will be critical to ensure expectations are met.

Overall, findings were consistent across the three study sites. We suspect this observation is related to the fairly standard implementation of the project across sites. For example, team members at each site have monthly site meetings, with representation from the central study site, providing the opportunity for regular project planning and implementation. All members of the patient council also meet quarterly to come together to discuss issues across the project as a whole. Further, sites share resources to help facilitate patient engagement (e.g., appreciation policy, templates for infographics to distribute study findings, patient engagement planning template). However, we presented at least one quote from a researcher/study staff member who indicated less positive experiences at their site. While we cannot draw strong conclusions from one data point, it is a reminder for project leads to pay close attention to the local context in which the project is being implemented. Turnover of study team members, prior and additional engagement experience of team members and study delays at sites could all contribute to more or less positive patient engagement experiences.

While the evaluation gained valuable insights into patient engagement in this large project in Canada, there were limitations. Overall, the sample size was small with notable differences in the survey response rates of patient partners (87.5%) and researchers/study research staff (34%). The lower response rate of these latter team members was disappointing and limits our ability to fully describe researcher and study staff experiences of patient engagement or address their challenges. We cannot know why more patient partners than researchers and study staff returned surveys, but postulate busy clinical schedules and perhaps survey timing (during the summer when vacations are at play). However, it is also possible that these team members did not feel engaged enough with patient partners to adequately contribute to the evaluation. While we purposively invited those team members who theoretically had opportunities to interact with the patient council, this did not result in a higher response rate. As the clinical trials in study sites roll out following pandemic delays, we are hopeful more opportunities will now naturally emerge for team members to interact and the end-of-study evaluation will have more equal response rates from all team members.

## Conclusion

Here, we presented the evaluation of patient engagement in a large research program in Canada. A recent systematic review noted the large degree of heterogeneity and diversity in frameworks for the evaluation of patient engagement, suggesting a local, co-developed approach to evaluation would be useful [15]. This was the approach taken by our team to co-design the evaluation of patient engagement in the project to date. Our data revealed that despite some challenges, team members recognized the value of patient engagement in research and agreed project decisions had been impacted by patient partner input. Ongoing communication was highlighted as an area for improvement, as well as earlier training and ongoing support for all team members, but particularly researchers and study staff. In response to evaluation data, the team has reinstated a quarterly newsletter and is using specific patient engagement planning templates [26] across study sites for all project activities. These tools should help make expectations clear for all team members and contribute to a positive patient engagement experience for all. Despite delays due to the pandemic, these evaluation data are critical for ongoing improvements in patient engagement and offer a baseline against which future evaluation data can be compared. Our co-designed evaluation approach and tools might also be useful to other research teams interested in evaluating their patient engagement activities.

### Supplementary Information

**Additional file 1.** Gripp2 Short form checklist.

**Additional file 2.** Evaluation survey instruments.

**Additional file 3.** The Do’s and Don’ts of Patient Partnership in Research infographic.

## Data Availability

The datasets used and/or analyzed during the current study are available from the corresponding author on reasonable request.

## Abbreviations

CIHR: Canadian Institutes for Health Research
CRTs: Cluster randomized trials
OHSN REB: Ottawa Health Science Network Research Ethics Board
PPC: Patient Partnership Council
ToR: Terms of reference
SPOR: Strategy for patient oriented research
