# Supplementary material for: “I think there has to be a mutual respect for there to be value”: Evaluating patient engagement in a national clinical trial on de-implementation of low value care
Source: Res Involv Engagem. 2023 Aug 26;9:70. doi: 10.1186/s40900-023-00483-w (PMC10463407; doi:10.1186/s40900-023-00483-w)
Supplement: Supplementary file 1 — Additional file 1. Gripp2 Short form checklist. [file 40900_2023_483_MOESM1_ESM.docx]

| **Section and topic** | **Item** | **Reported on page No** |
| --- | --- | --- |
| 1: Aim | The evaluation of patient engagement in research is understudied and under-reported, making it difficult to know what engagement strategies work best and when. In this report, we provide the results of an evaluation of patient engagement in a Canadian Institutes for Health Research (CIHR) Strategy for Patient Oriented Research (SPOR) Innovative Clinical Trial Multi-Year Grant (GRANT # MYG-158642). | 4 |
| 2: Methods | Two online surveys with a mix of closed and open items were created for the evaluation, one for patient partners and one for researchers/study research staff. The patient partner survey contained sections on motivation for becoming a patient partner, opinion on engagement to date in the project, opinion specifically on the PPC, with a final section for suggestions about engagement. | 6-7 |
| 3: Study results | The overall response rate was 46% (17/37). Seven of eight patient partners completed surveys (87.5%), while 10/29 (34%) researchers/study research staff returned surveys. We present patient partner evaluation data, followed by data from researchers/study staff; in the final section, we present data from the open-ended survey items asked of both groups of respondents. | 8-16 |
| 4: Discussion and conclusions | Most patient partners felt their contributions to the project were valued by researchers and study research staff, a motivator of ongoing engagement and satisfaction.^11,14^ Open comments revealed that a co-design approach and full inclusion on the research team was an integral part of the definition of ‘value’ for respondents, as well as ‘successful’ patient engagement, in line with the values of inclusion and mutual respect that underline Canada’s Strategy for Patient Oriented Research^15^, as well as best practices for patient engagement. | 16-20 |
| 5: Reflections/critical perspective | While there are high levels of willingness to be involved as a patient partner in research or to include patient partners on research teams, additional training and enhanced discussion about role expectations early in the process would help ensure a positive experience for all research team members. | 18-20 |
