# Supplementary material for: “I think there has to be a mutual respect for there to be value”: Evaluating patient engagement in a national clinical trial on de-implementation of low value care
Source: Res Involv Engagem. 2023 Aug 26;9:70. doi: 10.1186/s40900-023-00483-w (PMC10463407; doi:10.1186/s40900-023-00483-w)
Supplement: Supplementary file 2 — Additional file 2. Evaluation survey instruments. [file 40900_2023_483_MOESM2_ESM.docx]

**Patient Partner Experience Survey**

## **Motivations**

1. What was your motivation for becoming a patient partner on this research project?

## **Opinion on engagement in the research project**

Please indicate your thoughts on the following statements from 'Strongly Disagree' to 'Strongly Agree'.

1. Opinion on the engagement in the research project:
   - I was adequately oriented to the role of patient partner on this research project.
   - I understand my role and what is expected of me as a patient partner on this research project.
   - Researchers on the team value the experiences and suggestions that I share.
   - In general, my initial expectations of being a patient partner on this project have been met.
   - In general, I have the information and support I need to feel comfortable in my engagement.
   - Study staff value the experiences and suggestions that I share.
   - I would be willing to participate as a patient partner on a research project in the future.
   - Being a patient partner on this research project has been a valuable use of my time.
   - Overall, I have fulfilled my obligations as a patient partner on this research project.
2. If you wish, please provide any further or clarifying comments.

## **Opinion on the patient partnership council**

Please indicate your thoughts on the following statements from 'Strongly Disagree' to 'Strongly Agree'.

1. Opinion on the patient partnership council:
   - I am able to express my views freely on the patient partnership council.
   - I am satisfied with the frequency of patient partnership council meetings.
   - I feel that the input provided by the patient partnership council is considered by research project leads in decisions about the project.
   - I am satisfied with the length of the patient partnership council meetings.
   - A wide variety of views are valued on the patient partnership council.
   - Overall, I am satisfied with my membership in the patient partnership council.
2. If you wish, please provide any further or clarifying comments.

## **Final thoughts and suggestions about engagement in this project**

1. What would help keep you motivated for your involvement in this research project?
2. What has been the best part of your engagement on this research project to date?
3. What has been the most challenging part of your engagement on this research project to date (COVID-19 related or not)?
4. Please describe any impacts you have seen in the project as a result of patient partner input.
5. What could study staff and researchers do differently to improve the experience of patient engagement? Please share all ideas and suggestions for improvement.
6. How would you define *successful* patient engagement in this research project?
7. We want to better understand the idea of 'value' in the context of patient partner input. How can researchers/study staff show that they value patient partner contributions? What does this look like for you?
8. Finally, please select your province of residence:
   - Alberta
   - Newfoundland
   - Ontario

# **Research Team Experience Survey**

## **Opinion about the patient engagement in this research project**

Please indicate your thoughts on the following statements from 'Strongly Disagree' to 'Strongly Agree'.

1. Opinion about patient engagement in this research project:
   - The input of patient partners impacted the decisions of the research team.
   - Our patient partners are equipped to contribute to the research project.
   - I feel that my team and I are well prepared to work with patient partners on the research project.
   - Patient engagement is a good use of my time and resources.
   - I understand the role of patient partners on this research project.
   - I believe patient partners can improve the quality and outcomes of health research.
   - I would be willing to include a patient partner(s) in my future research projects.
   - So far, I am satisfied with my experience of patient engagement on this project.
   - In general, I have the information and support I need to feel comfortable engaging with patient partners in this project.
     - Please tell us if there is particular information, education or support that might help you to engage with patient partners.

## **Final open items about engagement**

1. At the start of the project, what were your expectations about engaging with patient partners on this project? Please tell us if these expectations have changed over time (for better or worse)?
2. Describe any impacts you have seen in the project as a result of patient partner input.
3. What could the patient partners or the patient partnership council do differently to improve your experience of patient engagement? Please share all ideas and suggestions for improvement.
4. How would you define successful patient engagement in this research project?
5. We want to better understand the idea of 'value' in the context of patient partner input. How can researchers/study staff show that they value patient partner contributions? What does this look like for you?
6. Please select the category that best describes your role on the project. *Your role in this project may be different than your general job description.
   - Researcher/Scientist
   - Study Research Staff
7. Finally, please select your province of residence:
   - Alberta
   - Newfoundland
   - Ontario
