# Supplementary material for: “I think there has to be a mutual respect for there to be value”: Evaluating patient engagement in a national clinical trial on de-implementation of low value care
Source: Res Involv Engagem. 2023 Aug 26;9:70. doi: 10.1186/s40900-023-00483-w (PMC10463407; doi:10.1186/s40900-023-00483-w)
Supplement: Supplementary file 3 — Additional file 3. The Do’s and Don’ts of Patient Partnership in Research infographic. [file 40900_2023_483_MOESM3_ESM.pdf]

The DO'S AND DON'TS of Patient Partnership in Research

DO make time to talk with us about your research – a conversation is all that's needed to begin the partnership

DON'T worry if your specific project has already started – it's never too late!

DO engage us early and be realistic in your expectations

DO hold Patient Partners to commitments as you would other project partners

DO explain time and financial constraints to us

DON'T forget that Patient Partners may not have the same work day hours and may perform their tasks in evenings or weekends

DO encourage us to help you with various project activities and tasks

DO have an open mind and be willing to hear our suggestions

DON'T hesitate to reach out if you are unsure about how to involve us

DON'T be afraid to tell us if our advice is not relevant/feasible given project constraints

DO give us specific tasks and be upfront about how much of our input can be used

DON'T ask for our input if it can't be incorporated in any way

DO provide an explanation of your expectations and check in to ensure they are understood

DO keep Patient Partners engaged in tasks, and make them meaningful

DON'T assume you need to figure this out on your own – co-development of patient partner roles is best practice for patient engagement

DO expect to hear personal stories about our experiences in the healthcare system; respect the confidentiality and facilitate opportunities for patients to tell their stories

DON'T assume we will focus only on our personal stories; our job is to offer the broader patient and public perspective

DO give us plenty of opportunity to ask questions and seek clarifications

DO make appropriate accommodations to build capacity and understanding

DON'T forget that Patient Partners' knowledge of your specialty is less than yours - please ask if we need clarification

Successful Partnership!

Developed by:
The De-implementing Wisely Patient Partnership Council
